# Death of Dr. Boling

**Published:** 1859-05

**Authors:** 


DEATH OF DR. BOLING.—It is with deep regret that we announce the death, after an illness of several years, of Dr. Wm. M. Boling, of Montgomery, Alabama. As a gentleman of high medical attainments and virtue, Dr. Boling had acquired an enviable reputation, and many valuable articles emanating from his pen are to be found in the periodical literature of this country. We sympathize with his numerous friends upon the loss of an honorable man and skillful physician.

